# Grafting based DNA methylation alteration of snoRNAs in upland cotton (*Gossypium* L.)

**DOI:** 10.1007/s12298-024-01469-y

**Published:** 2024-06-13

**Authors:** Mehmet Karaca, Ayse Gul Ince

**Affiliations:** 1 Department of Field Crops, Faculty of Agriculture, Akdeniz University, 07059 Antalya, Turkey; 2 Vocational School of Technical Sciences, Akdeniz University, 07059 Antalya, Turkey

**Keywords:** Differently methylated cytosine, Gene elements, Seedling vigor, Seed weight, WGBS

## Abstract

The effects of grafting in response to various biotic and abiotic stressors have been studied, however, the methylation status of small nucleolar RNA (snoRNA) genes in heterograft and homograft cotton needs investigation. This study was undertaken to determine grafting effects on DNA methylation of snoRNA genes in Upland cotton. Rootstocks used were Pima 3–79 (*Gossypium barbadense* acc. Pima 3–79) and Texas Marker-1 (*G. hirsutum* acc. TM-1), representing two different species with different fiber properties, adaptations, and morphologies. The methylation ratio and differently methylated cytosines (DMCs) of 10935 snoRNA genes in mature seeds of heterograft and homograft cotton samples were studied using the whole genome bisulfite sequencing method. Seedling vigor and seed weight were studied to investigate phenotype alterations that might be associated with altered methylation levels among grafts. Statistically significant DMC differences among gene elements of snoRNA genes and between homograft and heterograft cotton samples were identified in the absence of DNA sequence alterations. DNA methylation alterations of snoRNA genes associated with seedling vigor and 100 seed weight. The majority of snoRNA genes showed higher numbers of ^m^CG + ^m^CHG-DMCs with increased methylation levels in heterograft, while there were higher numbers of ^m^CG + ^m^CHG-DMCs with decreased methylation levels in homograft. Since snoRNAs regulate essential genes for plant growth and development and plant adaptation to different habitats or extreme environments, their altered methylation levels should be related with plant physiology.

## Introduction

The genus *Gossypium* contains more than 50 extant species, but three principal groups are of commercial importance. The first group includes *G. hirsutum* L., native to Mexico and Central America, which has the largest cotton production and adaptability, referred to as American Upland cotton. The second group includes *G. barbadense* L., native to the northern part of South America and extending into Mesoamerica and the Caribbean, which has the highest quality of fiber properties, referred to as Extra-Long Staple, American Pima, Sea Island, and Egyptian (Chen et al. 2020). The third group includes *G. herbaceum* L. and *G. arboreum* L., native to India and Eastern Asia, which have the shortest fiber among the commercial species. Upland and Pima are the main textile crops and important sources of edible oil, meal, and feed. Unfortunately, cotton production in the future seems to be at risk due to aridification, climate change, and new pest emergences (Karaca and Ince 2019). Furthermore, within the field crops, cotton is second only to maize in irrigation requirements.

Alternative production techniques for cotton would be useful for sustainable cotton production. One modern alternative production relies on the use of transgenesis-based cultivars such as Bt-transgenic insect-resistant cotton. However, while its use reduced the damage of *Lepidoptera* pests to some level, this approach has not reduced the negative pressures of aphids, sooty mites, and other pests (Wan et al. 2017). Another issue in cotton is related to quality issues of cotton fiber which have disadvantages of fineness, length, uniformity and strength in comparison to synthetic fibers. Therefore, cotton breeders are now faced with the challenges of not only improving fiber quality and yield but also sustainably advancing technologies in spinning. Future improvements in cotton cultivation and sustainability of production require not only the improvement of genomic resources and gene-editing tools but also the utilization of some other approaches such as transplanting, grafting, ratoon cotton cultivation, pruning, or carbon footprint reduction (Zhang et al. 2022).

A significant amount of the eukaryotic genome is transcribed into non-coding RNA molecules in comparison to protein coding regions producing mRNAs (Bratkovic et al. 2020). Among the non-coding RNAs, small nucleolar RNAs (snoRNAs), an abundant class of 50–300 nucleotide trans-acting RNAs associated with small nucleolar ribonucleoprotein particles (snoRNPs), play significant roles in post-transcriptional modifications of ribosomal RNAs (rRNAs), transfer RNAs (tRNAs), and small nuclear RNAs (snRNAs). In addition to these functions, increased numbers of studies are reporting novel functions of snoRNAs (Chen et al. 2021; Fernandes et al. 2023; Wang et al. 2023). snoRNAs are encoded by intronic regions of both protein coding and non-protein coding genes and organized in polycistronic clusters (Bratkovic et al. 2020). Modifications of transcribed snoRNAs occur in the nucleolus while Cajal body specific RNAs (scaRNAs) mainly function in the Cajal bodies, sub-nuclear structures that are physically and functionally associated with the nucleolus (Wang et al. 2023).

The main aim of grafting is to combine desired traits from two different plants by joining the top part of a plant (scion) onto another plant with a stem and roots (rootstock). Although grafting has been commonly used in horticulture for a long time, the biological mechanisms driving rootstock-induced alterations of the scion phenotype remain unknown (Avramidou et al. 2015; Liu et al. 2023a, b). Although cotton does not belong to crop species that are commercially grafted, such as cucumber, tomato, and watermelon, grafting in cotton provides alternative ways to investigate the genetic and epigenetic variations in *Gossypium* (Karaca et al. 2020; Heilsnis et al. 2021). The application of grafting in cotton included improving stress tolerance, increasing nutrient and water use efficiency, and cultivating cotton perennially for high yield and production (Garcia-Lozano et al. 2020; Li and Zhao 2021). It may be useful in cotton breeding for some reasons such as conserving and propagating special germplasms, utilizing heterosis, breeding new varieties, improving the survival rate of transplanted plantlets, and accelerating resistance gain for biotic and abiotic stress factors (Spano et al. 2017; Cerruti et al. 2021; Ye et al. 2023). Furthermore, grafting could be used in cotton physiology and molecular biology research, such as the mechanism underlying root-shoot communication (Chen et al. 2022; Heilsnis et al. 2021; Jeynes-Cupper and Catoni 2023; Yang et al. 2023).

In this study, grafting experiments were made between Pima 3–79 (*G. barbadense* accession Pima 3–79) and Texas Marker-1 (*G. hirsutum* accessions TM-1). Heterograft, homograft, and ungraft control plants were grown to maturity, and seed samples were obtained to investigate the DNA methylation status of snoRNA genes. Furthermore, tests of seedling vigor and 100 seed weights in graft and control samples were conducted to reveal the phenotypic effects of altered DNA methylation status of snoRNA genes.

## Materials and methods

### Plant materials

In grafting experiments, accession of Pima cotton, Pima 3–79 (*G. barbadense* L.), and accession of Upland cotton, Texas Marker-1, TM-1 (*G. hirsutum* L.) were used as the scion, rootstock, and ungrafted control. Seeds of TM-1 and Pima 3–79 treated chemicals against fungal diseases were sown in 350 mL pots filled with peat moss (Klasmann-Deilmann, Germany) on April 17, 2021, and April 22, 2022. Two seeds per pot were sown, and the pots were submerged in a field at Akdeniz University in Türkiye. In this way, healthy and homogenic seedlings of TM-1 and Pima 3–79 were obtained. Seedlings at the two leaf stages were brought to a laboratory and acclimatized for 3 days prior to grafting experiments. Ten milliliters of fertilizer solution were provided to seedlings three days before grafting and five days after grafting (Karaca et al. 2020).

### Grafting experiments

Throughout this study, TK was referred to as the ungrafted (ungraft control) TM-1. PT was the graft of Pima 3–79—TM-1 and TT was the graft of TM-1—TM-1. Homograft and heterograft were referred to as TT and PT, respectively. The grafting method of Karaca et al. (2020) was used in all grafting experiments. A seedling, used as a scion, was cut with a sterile razor blade 4–5 cm below the apex, and another seedling, used as a rootstock, was cut vertically below the cotyledonary node to a depth of 3 cm to have a deep wedge ‘V’ shape. The stem end of a scion was prepared in a way so that when it was inserted into a stem of rootstock, it could fit well, and the graft union was secured with Parafilm. Graft and control seedlings were grown for 15 days in a growth tunnel and then transferred to a greenhouse at Akdeniz University. The experimental design was a randomized, complete plot with three replications. There were at least thirty plants in each replication, and all plants received standard agronomic practices throughout the growing seasons as described in Karaca et al. (2020).

### Seedling vigor index (SVI) and 100 seed weight (100SW)

In this study, seedling vigor values, which indicate the ability of seeds to emerge rapidly from soil, were determined based on the average weight of hypocotyls obtained after 96-h germination period at 28 °C for 16 h and 25 °C 8 h at dark in plastic dishes in a germination chamber. One hundred seeds were germinated between 4 × germination papers. There were three replications for each sample.

$$Average{\, } Weight {\, }of{ \,} Hypocotyls \left( g \right) = Weight \left( g \right) { \,}of{ \,} hypocotyls { \,} obtained {\,}from {\, }100{ \,} seeds$$

$$Germination{ \,} No = \frac{{Number { \,}of {\, }seeds {\, }with {\, }at{\, } least {\, }5 {\text{mm}} {\, }hypocotyl \left( {No} \right) }}{{Number{ \,} of{\, } seeds{ \,} tested}}$$

$$Seedling { \,}Vigor{\, } Index \left( {SVI} \right) = Average{ \,} Weight {\, }of { \,}hypocotyls \left( g \right) \times Germination{ \,} No$$

The weights of one hundred hand-ginned seeds were weighed to determine 100SW values using three replications. Differences among measured values of seedling vigor index and 100SW values were assessed using analysis of variance (ANOVA) coupled with Tukey–Kramer HSD by utilizing the JMP Statistical Discovery Software Version 8.0 (SAS). Statistically significant differences were assessed at the *p* < 0.05 significant level.

### Genomic DNA extraction, library preparation, and cluster sequencing

Genomic DNA samples were extracted from six individual hand-uncoated seeds collected from ten different cotton grafts or controls using a DNA extraction method (Karaca and Ince 2023a). The extracted DNA samples were further purified using a DNA extraction kit. There were two biological replications for each DNA sample. Cotton genomic DNA was fragmented by sonication to 200–300 bp with a Covaris LE220 sonicator after adding 0.5% (w/w) lambda phage DNA (48502 bp, Promega, Madison, WI, USA). Fragmented genomic DNA and lambda phage DNA were treated with bisulfite to convert unmethylated cytosines to uracils while retaining those cytosines that are methylated. Reactions were using the EZDNA Methylation-Gold kit following the manufacturer's instructions (Zymo Research, Irvine, CA, USA). The sequencing libraries were prepared using the Accel-NGS Methyl-Seq DNA library kit (Swift BioSciences, Ann Arbor, MI, USA) according to the library protocol for Illumina platforms. Library preparation and cluster sequencing analysis were performed by Macrogen Corp., on an Illumina Nova Seq 6000 with 150 Gb of 151-bp Paired-End sequencing.

### Data processing of whole genome bisulfite sequencing (WGBS)

Twenty base pairs were trimmed from the 3′- ends of forward and 5′- ends of reverse sequences to eliminate the majority of the adaptase tails, and those reads shorter than 20 bp were discarded using Trim Galore (Krueger 2021). Raw and filtered reads obtained from each sample were compared using FastQC Screen to confirm the quality increase (Wingett and Andrews 2018). Total read bases ranged from 60.31 to 69.13 Gbp among samples. In this study, reads with a Phred quality score greater than or equal to 30 (Q30) were utilized. The cleaned reads in FASTQ format were aligned to the *G. hirsutum* TM-1 reference genome (GCF_007990345.1) using BSMAP based on the short oligo alignment program (SOAP) piped with SAMtools view-bs to obtain mapped bam files (Li et al. 2009).

The evaluation of the quality of the sorted alignment data was performed using Qualimap 2.2 (Okonechnikov et al. 2016). Further analysis used uniquely mapped reads after sorting them according to chromosomes and genomic positions. Cleaned reads were used in the methyratio.py script to extract methylated and unmethylated cytosines in the three-sequence contexts of CG, CHG, and CHH (where H is any base other than G), along with their coverage profile values. During data preprocessing, low coverage and high coverage bases were filtered using lower and higher cutoff values, 15 and 500, respectively. A lower read cut-off of 15 means that bases with coverage below 15 × were discarded because a high enough read coverage would increase the power of the statistical tests. The estimation of bisulfite conversion rate was determined by adding 0.1–5% (w/w) unmethylated lambda phage DNA before bisulfite treatment. The bisulfite conversion ratio (BCR, %) of the lambda DNA was calculated using the following formula:

$$\text{BCR }= \frac{\text{No } \, \text{unmethylated reads} }{\text{No methylated reads}+\text{ No unmethylated reads} } \times 100$$

### Methylation percentage values and differently methylated cytosines (DMCs)

Genomic positions of snoRNA gene body regions along with 5000 bp upstream and 2000 bp downstream regions were extracted from WGBS data, and their weighted mean methylation percentage values were calculated using the given cut-off of 15 × minimum coverage by utilizing Defiant software (Condon et al. 2018). A *p* value between samples A and B was calculated by Fisher's exact test using the following formula:

$$p= \frac{\left(\text{mCA}+\text{mCB}\right)!\left(\text{CA}+\text{CB}\right)!\left(\text{mCA}+\text{CA}\right)!\left(\text{mCB}+\text{CB}\right)! }{\text{mCA}!\text{mCB}!\text{CA}!\text{CB}!\left(\text{mCA}+\text{mCB}+\text{CA}+\text{CB}\right)! }$$

where ^m^C = number of 5-methyl cytosine and C = number of cytosine.

Differently methylated cytosines (DMCs) were defined as cytosines that had $p$ < 0.05 and a methylation difference > 20% between control and graft methylation rates. Only was considered DMCs found in all samples to make more rigorous detection of methylation changes. The number of ^m^CG—^m^CHG -DMCs with increased and decreased methylation levels was compared between TK vs PT and TK vs TT (Table 1).

**Table 1:** Primer pairs and loci used in amplification reactions of DNA samples

ID	Forward primer sequences (5′➔3′)	Reverse primer sequences (5′➔3′)
KM16	GAGAAGATTGATTATTTGAAGGAYAAG	RATATCAAARAARACAACATTTCRATT
KM17	AGATGGAAAYGAGTTTGTTATYTYTAA	AARTRATTCTTRTCTCTRTAACCTCAT
KM18	CTTYTTTYGTYTTAATTGGTTTYAYTA	AATAACTCCTCACATTARAACCATCAC
KM19	TGTTAGTGGTAYYATTTATAYYGATG	AATTACATTTTCACACAATTACCCTRT
KM20	TAYAGAATTYGGTGAAYTTTATYTYTT	TTTARAATCACARCAAATCAACAATAR
KM21	AYTTATYGAAAAATAYAAGTTGAATGG	TAATARACATRATTRTTTTACCCRATT
KM22	YYTTATTGGATGTAGTAYAAGGATGTT	CTGCAACCTTCTTTTAACTRTATRAR
KM23	CTGATYTGGTTYATTTTATYYTAYAGA	ATACARATAACTCCTCACATTTRATCC
KM24	GGTAYGATAAAGTGGTTAAYATTYYTA	RAAAATCAATRAATTCRTAACCTAAAA
KM25	TTGCCTCACAAAACAAGCAAAC	GCCATCATTATCATCAACAACG
KM26	CCCCAAAACTATTTGTATTGTTCC	TGAAAACCGGAAGGAATGAG
KM27	GCCGTAACCTGTGGTCAAGT	AGCTGGTGAACTTTCCATGC
KM28	CCGGGTCGAATTTAAACAAA	GAGCCGGTGGGTAAGTGTAG
KM29	ACAGATTAGGAATGAGAAACTGTCATC	ATGCTTAGCCAATTCCATGC
KM30	AGCGACGGAGACAAATAAGG	AAGAGGAAGGGGACCAAAGA
IN01	GGATTGGTTTTYTYAYATYATATTTTA	CGTTGTTTRTTCTTRCATTARTAATTT
IN02	AATGTGTTGTTATTGGTTGAGTGTAAA	TAATTTTCTARRCCTCATARTRRTAAC

*: R indicated a purine (A or G), and Y indicated a pyrimidine (C or T)

### Touchdown polymerase chain reactions (Td-PCRs)

Twenty-four genomic DNA samples, including 8 TT and 8 PT, along with 8 ungraft TK control were used to investigate the DNA sequence alterations that might be caused by grafting. Touchdown polymerase chain reactions (Td-PCR) were conducted in a 25 μL reaction volume (Ince and Karaca 2021). Agarose gel electrophoresis and visualization of PCR products were performed as described in Karaca and Ince (2023a). DNA markers on the agarose gels were scored for identification of DNA loci alteration. The presence or absence of a marker between a graft and its control counterpart was scored. Alterations were defined as the proportion of sites on the chromosome at which two randomly chosen copies differ in DNA sequence using the formula h = 1 − ∑x^2^i, h = 1 − ∑xi^2^, where xi is the frequency of the ith allele. The value h reflects the underlying mutation (alteration) rate (Karaca et al. 2020). Furthermore, amplified bands were digested using the *Msp*I restriction enzyme to detect any sequence alteration at *Msp*I recognition sites among samples (Karaca et al. 2019).

## Results

### Seedling vigor and seed weight in graft

**Fig. 1:**
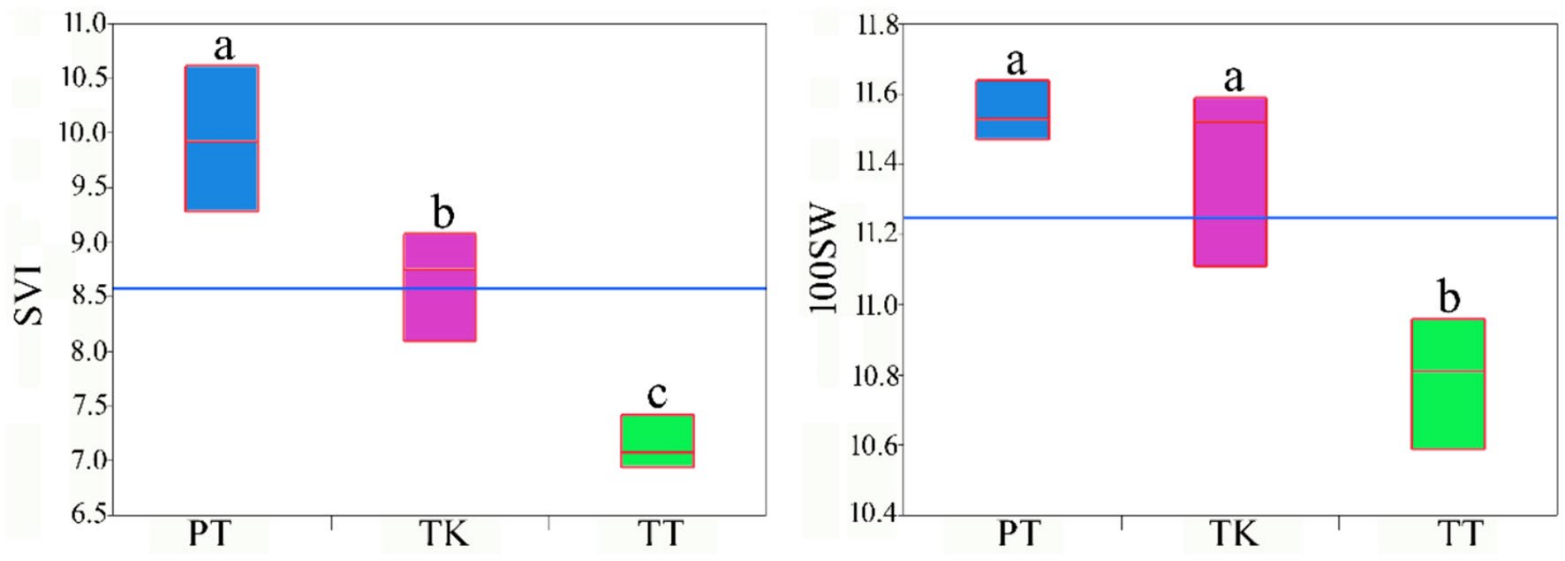
Seedling vigor index (SVI) and 100 seed weight (100SW) values among control and graft samples. TK: TM-1 (ungraft), PT: Pima 3–79—TM-1 (PT), and TT: TM-1—TM-1 (TT). Please note that levels not connected by the same letter are significantly different (*p* < 0.05)

As an important research topic in crops, the mechanisms driving rootstock induced changes in scion physiology and genomics remain poorly understood. To investigate the effects of grafting on scion physiology, seedling vigor index (SVI) and 100 seed weight (100SW) values were determined using seeds obtained from PT [Pima 3–79–TM-1], TT [TM-1—TM-1] and ungraft TM-1 control (TK). Results revealed that seeds of PT had a significantly increased SVI in comparison to ungraft TK and TT (*p* < 0.05). The 100SW values were different (*p* < 0.05) between TT and PT. The values of 100SW of PT were higher than TT (Fig. 1). Increased SVI and 100SW in PT indicated that Pima rootstock positively affected SVI and 100SW of Upland scion. It is known that rootstocks are able to change the physiological environments in scions such as water potentials, concentrations of mineral elements, and hormones (Cookson and Ollat 2013; Kundariya et al. 2020; Heilsnis et al. 2021; Liu et al. 2023a, b). Larger seeds are supposed to have higher SVI, but this was not the case seen in ungraft (TK), which had similar values of 100SW with PT.

### snoRNA genes in upland cotton

The reference genome utilized in the present study had 10935 snoRNA genes, corresponding to 102 snoRNA types. Among snoRNAs, snoR71 had 10348 copies, while snoR145, Z159/U59, snoR113, snoR138, snoR126, snR60/Z15/Z230/Z193/J17, R66, U19, SNORD36, Z278, R32/R81/Z41, snoR136, snoR116, snoR28, snoR127, Z152/R70/R12, U54, U83, snoR97, snoR100, and R72 had two copies. snoRNAs R32/R81 had the second highest copies (93 copies), followed by U3 (30 copies), snoR114 (24 copies), and SNORD14 (20 copies). The remaining 78 snoRNAs had copies ranging from 3 to 17. Compared to other eukaryotes, plants have more snoRNA genes due to polyploidy and chromosomal rearrangements. Chen et al. (2021) reported a total of 3626 snoRNAs, 1712 of which were C/D snoRNAs, 86 were H/ACA snoRNAs, and 1828 were orphan snoRNAs in the rubber tree (*Hevea brasiliensis*). Compared to crops, *G. hirsutum* had many snoRNA genes (Bhattacharya 2016; Han et al. 2022). The large snoRNA family of cotton is due to a high degree of gene duplication, potential gene redundancy, and a higher number of rRNA methylations (Fernandes et al. 2023). The selection of snoRNAs in cotton is therefore an ideal model for investigating the effects of grafting on non-coding genes.

The distribution of snoRNAs varied among chromosomes, subgenomes, and strands of *G. hirsutum* acc. TM-1 genome as shown in Table 2. The mean length of snoRNAs was 106.66 bp, ranging from 64 to 225 bp. Each chromosome of *G. hirsutum* contained snoRNAs, but chromosome A08 of subgenome A had the highest number of snoRNAs per mega base (mb) while chromosome A05 of subgenome A had the lowest number of snoRNAs per mb. The occurrence of snoRNAs on plus ( +) and minus (–) strands also showed variations in subgenome A (Table 2). Chromosome D01 of subgenome D had the highest number of snoRNAs per mb, while chromosome D05 of subgenome D had the lowest number of snoRNAs per mb. Based on the results of this study, it can be stated that subgenome A had more snoRNAs (8042) in comparison to subgenome D (2893). The presence of higher number of snoRNAs in subgenome A was not due to size of subgenome A. Subgenome A had a snoRNA gene density of 5.65 while subgenome D had a snoRNA density of 3.46.

**Table 2:** Distribution of number of snoRNA genes on chromosomes, subgenomes and strands of TM-1

Chromosome		No snoRNAs on plus ( +) strand		No snoRNAs on minus (-) strand		Total snoRNAs and density	
Chr ID	Length (mb)	No	No/mb	No	No/mb	No	No/mb
A01	119.76	389	3.25	377	3.15	766	3.15
A02	108.14	267	2.47	288	2.66	555	2.66
A03	113.69	313	2.75	308	2.71	621	2.71
A04	89.18	247	2.77	250	2.80	497	2.80
A05	111.10	219	1.97	233	2.10	452	2.10
A06	128.20	426	3.32	406	3.17	832	3.17
A07	98.90	277	2.80	314	3.17	591	3.17
A08	127.50	386	3.03	434	3.40	820	3.40
A09	85.34	187	2.19	185	2.17	372	2.17
A10	118.18	335	2.83	321	2.72	656	2.72
A11	124.18	310	2.50	271	2.18	581	2.18
A12	109.47	295	2.69	300	2.74	595	2.74
A13	111.65	352	3.15	352	3.15	704	3.15
A subG	1445.29	4003	2.77	4039	2.79	8042	5.56
D01	65.21	129	1.98	153	2.35	282	2.35
D02	72.19	139	1.93	154	2.13	293	2.13
D03	54.96	98	1.78	123	2.24	221	2.24
D04	58.23	100	1.72	136	2.34	236	2.34
D05	66.48	44	0.66	58	0.87	102	0.87
D06	66.68	134	2.01	130	1.95	264	1.95
D07	59.44	78	1.31	87	1.46	165	1.46
D08	69.43	141	2.03	154	2.22	295	2.22
D09	54.45	67	1.23	71	1.30	138	1.30
D10	68.09	120	1.76	126	1.85	246	1.85
D11	72.82	117	1.61	86	1.18	203	1.18
D12	63.26	90	1.42	104	1.64	194	1.64
D13	65.10	122	1.87	132	2.03	254	2.03
D subG	836.33	1379	1.65	1514	1.81	2893	3.46

Chr: Chromosome, A subG: *G. hirsutum* subgenome A, subG: *G. hirsutum* subgenome D

### Whole-genome bisulfite sequencing (WGBS)

In this study, WGBS method was used to locate methylated cytosines by treating genomic DNA of seeds from each grafted and ungrafted cotton with sodium bisulfite before performing a next generation DNA sequencing study. The average throughput depth of sequencing among 6 samples ranged from 48.8 × to 56.2x. In all analyses at least 15 × coverage of targeted snoRNAs were utilized to improve the power of the analysis with the use of two biological replicates (Ziller et al. 2015). In the last decade, WGBS has become the gold standard for studying genome-wide methylation at single base resolution. One of the important aspects of the WGBS method is to obtain reliable conversion of unmethylated cytosines (Liu et al. 2023a, b). Results revealed that higher conversion rates (%), which were the number of clean unmethylated reads mapped to lambda DNA over the number of total clean methylated and unmethylated reads mapped to lambda DNA, were obtained, ranging from 99.6499 to 99.6633%. This showed that DNA samples were successfully treated with bisulfite. All snoRNAs of the reference genome utilized in the present study were completely mapped with WGBS data. Based on the high coverage and high-quality reading, this study once again confirmed that WGBS is a reliable technology to detect DNA methylation on a genome-wide scale at single-base resolution (Liu et al. 2023a, b). WGBS has been applied to many plant species, including cotton (Ziller et al. 2015; Cao et al. 2016; Perrin et al. 2020; Cerruti et al. 2021; Chen et al. 2022; Jeynes-Cupper and Catoni 2023).

### Methylated cytosines in snoRNA gene elements

Results revealed that mature seed DNA sequences of cotton snoRNAs methylated significantly. The principal methylation context was ^m^CG in all samples and gene elements. The means of methylation ratios ranged from 72.53 to 92.35% for ^m^CG, from 48.47 to 82.09% for ^m^CHG and from 13.38 to 15.09% for ^m^CHH (Table 3). The overall study revealed that methylation levels in snoRNAs were alike the methylation levels of the whole genome in terms of the occurrences of ^m^CG, ^m^CHG and ^m^CHH in other plant models such as soybean, barrel clover, maize, *Arabidopsis*, tomato, watermelon, among others (Gardiner et al. 2018; Xu et al. 2019; Cerruti et al. 2021; Villagomez-Aranda et al. 2021; Liu et al. 2023a, b).

**Table 3:** Methylation ratios (%) of snoRNA gene regions among grafted and control cotton samples

Gene regions	Methylation content	TM-1 (Ungraft, TK)	TM-1—TM-1 (Homograft, TT)	Pima 3–79—TM-1 (Heterograft, PT)
UPS (-5 kbs)	^m^CG	92.16(± 1.09)	91.74(± 1.21)	92.35(± 1.06)
GB		75.86(± 2.24)	72.53(± 5.12)	77.53 (± 1.76)
DWN (+ 2 kbs)		91.50(± 1.26)	90.90(± 1.71)	91.49(± 1.26)
UPS (-5 kbs)	^m^CHG	81.92(± 1.02)	81.19(± 0.45)	82.09(± 0.92)
GB		52.99(± 3.97)	48.47(± 7.83)	52.92(± 4.48)
DWN (+ 2 kbs)		81.89(± 1.45)	81.12(± 1.12)	81.85(± 1.58)
UPS (-5 kbs)	^m^CHH	14.55(± 0.21)	14.48(± 0.23)	14.71(± 0.11)
GB		13.55(± 0.36)	13.38(± 0.16)	13.88(± 0.26)
DWN (+ 2 kbs)		14.94(± 0.64)	14.90(± 0.27)	15.09(± 0.25)
UPS (-5 kbs)	^m^CG + ^m^CHG + ^m^CHH	62.88(± 1.01)	62.47(± 1.25)	63.05(± 1.02)
GB	^m^CG + ^m^CHG + ^m^CHH	47.47(± 2.13)	44.79(± 4.47)	48.11(± 1.83)
DWN (+ 2 kbs)	^m^CG + ^m^CHG + ^m^CHH	62.78(± 1.24)	62.30(± 0.64)	62.81(± 0.29)
Overall	^m^CG + ^m^CHG + ^m^CHH	57.71(± 1.78)	56.52(± 1.79)	57.99(± 0.70)

*UPS*: Upstream, *DWN*: Downstream, GB: Gene body regions of snoRNAs in *G. hirsutum* L.

Three gene elements of snoRNAs were studied to reveal any DNA methylation differences associated with grafting in *G. hirsutum* cotton. Results revealed that methylation levels of upstream (UPS) and downstream (DWN) regions were similar among TT, PT and ungraft TK in ^m^CG, ^m^CHG and ^m^CHH (Table 3). On the other hand, the UPS and DWN regions of snoRNA genes had significantly higher methylation levels in ^m^CG, ^m^CHG and ^m^CHH in comparison to gene body (GB) regions (Table 3). It was noted that methylation status of snoRNAs in cotton seeds showed similar patterns with those protein coding genes in plants in which methylation levels of GB regions were usually lower than that of UPS and DWN regions (Liu et al. 2023a, b). This similarity was surprising since GB of snoRNA genes and GB of protein coding regions differ in their lengths and functions. For instance, while snoRNA genes are usually 50–300 nucleotides trans-acting RNAs, protein coding genes vary greatly in length but usually longer than snoRNAs. snoRNA genes do not encode proteins meaning that they have more opportunity to accumulate mutations.

Comparison studies revealed that there were not statistically significant differences in ^m^CG and ^m^CHG and ^m^CHH among TT, PT and ungraft TK. As it can be seen in Table 3, GB regions showed more methylation differences in ^m^CG and ^m^CHG contexts in comparison to UPS and DWN regions. However, due to existence of variations between biological replications, these differences were not statistically different (*p* < 0.05). On the other hand, ^m^CG and ^m^CHG levels of UPS and DWN regions showed little variations between biological replications. It was noted that there were methylation level differences calculated using 10 × and 15 × coverage as reported earlier (Ziller et al. 2015).

The data presented in Table 3 showed overall methylation differences, therefore, an essential task was required to detect the methylation differences using either at locus-by-locus or several loci within a DNA region. Differential methylation can occur either at single cytosine (DMC) or at several loci within a region, resulting in differentially methylated regions (DMRs). However, the GB of snoRNAs studied had an average length of 106.6 bp, thus, short stretches limited obtaining the groups of cytosines into regions with a specific segmentation method such as any of DMR detection (Condon et al. 2018).

### DMCs in snoRNA gene elements

The identified DMCs were annotated according to their chromosome positions and were assigned to a specific snoRNA gene using the annotation information of the TM-1 reference genome. There was a total of 11,063 DMCs when ungraft TK and TT were compared (TK vs TT). There were 11290 DMCs when ungraft TK and PT were compared (TK vs PT) for ^m^CG + ^m^CHG contents. However, there were no DMCs detected for ^m^CHH content in TK vs TT and TK vs PT. Total DMCs consisted of the numbers of ^m^CG + ^m^CHG with decreased and increased methylation. Increased or decreased methylation levels were based on the comparison of the numbers of DMCs between ungraft TK and TT or PT showing decreased or increased methylation differences (Table 4). It is known that the numbers of DMCs detected by most methods increase as the sequencing coverage or the numbers of replicates increase (Condon et al. 2018; Piao et al. 2021; Villagomez-Aranda et al. 2021).

**Table 4:** Differently methylated cytosines (DMCs) in snoRNA genes among grafts and control

Comparisons	No DMCs	No increased DMCs	No decreased DMCs
Methylation Content (^**m**^CG + ^**m**^CHG)			
TM-1 (ungraft) vs [Pima 3–79–TM-1]	11290	5420	5870
TM-1 (ungraft) vs [TM-1—TM-1]	11063	5213	5850
Methylation Content (^**m**^CG)			
TM-1 (ungraft) vs [Pima 3–79 –TM-1]	1684	782	902
TM-1 (ungraft) vs [TM-1—TM-1]	1625	698	927
Methylation Content (^**m**^CHG)			
TM-1 (ungraft) vs [Pima 3–79 –TM-1]	9606	4638	4968
TM-1 (ungraft) vs [TM-1—TM-1]	9438	4515	4923
Gene Region (Upstream) ^**m**^CG + ^**m**^CHG			
TM-1 (ungraft) vs [Pima 3–79–TM-1]	8422	4019	4403
TM-1 (ungraft) vs [TM-1—TM-1]	8306	3906	4400
Gene Region (Gene Body) ^**m**^CG + ^**m**^CHG			
TM-1 (ungraft) vs [Pima 3–79–TM-1]	100	44	56
TM-1 (ungraft) vs [TM-1—TM-1]	101	50	51
Gene Region (Downstream) ^**m**^CG + ^**m**^CHG			
TM-1 (ungraft) vs [Pima 3–79 –TM-1]	2768	1357	1411
TM-1 (ungraft) vs [TM-1—TM-1]	2656	1257	1399

The results showed that although the numbers of ^m^CG + ^m^CHG-DMCs with decreased methylation were similar when ungraft TK vs PT, and TK vs TT were compared, the numbers of ^m^CG + ^m^CHG-DMCs with increased methylation were significantly different (5420 vs 5213) indicating that heterografting caused an increase in the numbers of ^m^CG + ^m^CHG-DMCs with increased methylation level (Table 4). It was noted that when the overall numbers of increased and decreased ^m^CG + ^m^CHG-DMCs were compared, both types of grafts had higher numbers of ^m^CG + ^m^CHG-DMCs with decreased methylation levels than ^m^CG + ^m^CHG-DMCs with increased methylation levels indicating that grafting caused an increase in the number of demethylated DMCs (Table 4). Further studies revealed that although homografts and heterografts had a higher numbers of ^m^CG + ^m^CHG-DMCs with decreased methylation levels, the magnitude of increased DMCs in TT was lower than in PT (Table 4).

Results revealed that numbers of ^m^CHG-DMCs were approximately 5 times higher than those of ^m^CG-DMCs. The numbers of DMCs in ungraft TK vs PT had a higher numbers of ^m^CHG-DMCs with increased methylation levels than ungraft TK vs TT. The numbers of ^m^CG-DMCs with increased methylation levels in ungraft TK vs PT were higher than in ungraft TK vs TT (Table 4). This indicated that grafting affected methylation levels by influencing probably the activities of domains rearranged methyltransferase 1 (DRM1) and (DRM2) and directed by small interfering RNAs (siRNAs) (Erdmann and Picard 2020; Agius et al. 2023). Differences in the numbers of ^m^CG + ^m^CHG-DMCs among three gene elements were studied, and results revealed that among the gene elements, UPS regions contained the highest numbers of ^m^CG + ^m^CHG-DMCs, followed by DWN regions but the lowest numbers were observed in GB regions (Table 4). The lower number of methylation levels GB was mainly due to relatively shorter length of gene body regions. DMCs in ungraft TK vs PT had higher numbers of ^m^CG + ^m^CHG-DMCs with increased methylation levels than ungraft TK vs TT in UPS and DWN regions (Table 4). There were similar trends in the numbers of ^m^CG + ^m^CHG with decreasing methylation levels between UPS and DWN regions in TT and PT. Differing from UPS and DWN regions, GB regions had more ^m^CG + ^m^CHG with increasing methylation levels in ungraft TK vs TT than ungraft TK vs PT. On the other hand, GB regions had lower numbers of ^m^CG + ^m^CHG with decreasing methylation levels in ungraft TK vs TT than ungraft TK vs PT. These results indicated that among the gene elements, GB regions showed different responses to grafting in comparison to UPS and DWN regions in cotton.

### Differentially methylated cytosines (DMCs) in subgenomes and chromosomes

The methylation levels of A and D subgenomes in three different gene elements were studied. Results revealed that numbers of ^m^CG + ^m^CHG-DMCs with increased methylation levels were higher in the A subgenome than D subgenome when ungraft TK vs PT, and TK vs TT were compared in both UPS and DWN regions. However, there were similar numbers of ^m^CG + ^m^CHG-DMCs with decreased methylation levels between TT and PT in UPS and DWN regions. However, compared to UPS and DWN regions, GB regions of A subgenome contained higher numbers of ^m^CG + ^m^CHG-DMCs with decreased methylation levels in ungraft TK vs TT than in ungraft TK vs PT. On the other hand, the methylation level differences among gene elements of snoRNA genes in D subgenome were similar between TT and PT. Based on these findings, it was suggested that grafting caused more methylation alterations in A subgenome than D subgenome.

**Fig. 2:**
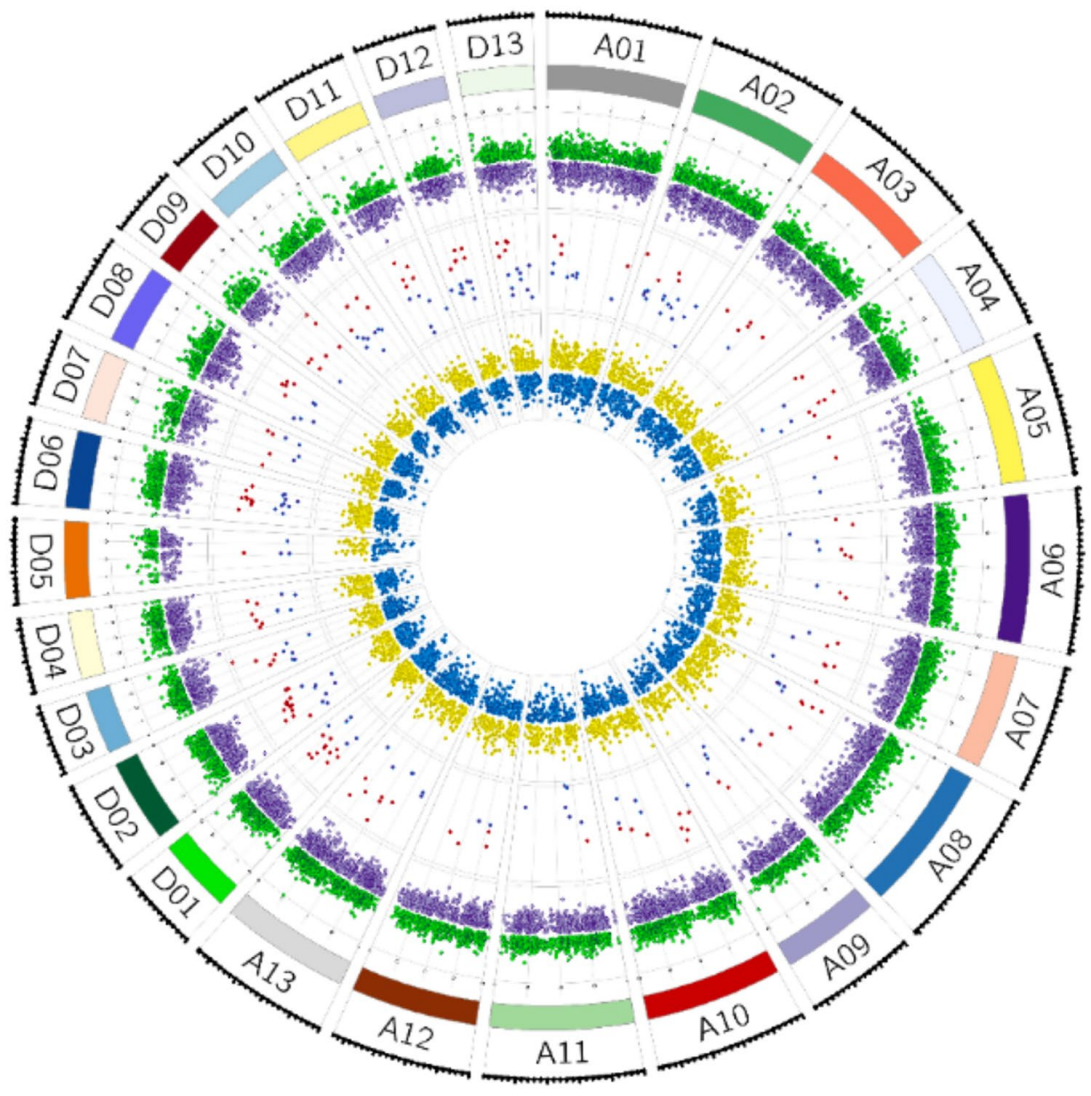
Locations of snoRNAs and ^m^CG + ^m^CHG-DMCs with increased and decreased methylation levels in TK (ungraft) vs heterograft [Pima 3–79—TM-1] and TK vs homograft [TM-1—TM-1] throughout cotton chromosomes and subgenomes. The outermost circle represented the numbered chromosomes of *G. hirsutum* A and D subgenomes, and chromosome sizes are marked by a scale plate. Please note that colored dots towards the center represent the number of ^m^CG + ^m^CHG-DMCs with increased and decreased methylation in upstream (UPS, outer circle), gene body (GB, middle circle), and downstream (DWN, central cycle). Green and lilac (UPS), red and navy GB, and yellow and blue represented increased or decreased ^m^CG + ^m^CHG-DMCs, respectively. DMCs: Differently Methylated Cytosines

The distributions of ^m^CG + ^m^CHG-DMCs with decreased and increased methylation levels among chromosomes were shown in Fig. 2. Chromosome A08 (739) and A09 (351) in the A subgenome and chromosome D02 (420) and D05 (351) in D subgenome had the highest and lowest number of ^m^CG + ^m^CHG-DMCs in ungraft TK vs PT, respectively. On the other hand, chromosome A06 (715) and A09 (353) in A subgenome and chromosome D08 (34) and D05 (145) in D subgenome had the highest and lowest number of ^m^CG + ^m^CHG-DMCs in ungraft TK vs TT, respectively. Furthermore, chromosomes of both subgenomes showed differences in the number of ^m^CG + ^m^CHG-DMCs with increased and decreased methylation levels. When numbers of ^m^CG + ^m^CHG-DMCs with increased and decreased methylation levels were compared, chromosomes A03, A06, A13, D09, D10, and D11 had relatively higher numbers of ^m^CG + ^m^CHG-DMCs with deceased methylation levels in ungraft TK vs PT while A01, A07, A08, D01, D04, and D06 had higher numbers of ^m^CG + ^m^CHG-DMCs with deceased methylation levels in ungraft TK vs TT. On the other hand, chromosome D13 had the highest number of ^m^CG + ^m^CHG-DMCs with increased methylation levels in ungraft TK vs TT while A11, D03, and D11 had higher numbers of ^m^CG + ^m^CHG-DMCs with increased methylation levels in ungraft TK vs TT.

### Differentially methylated cytosines (DMCs) in snoRNA genes

**Fig. 3:**
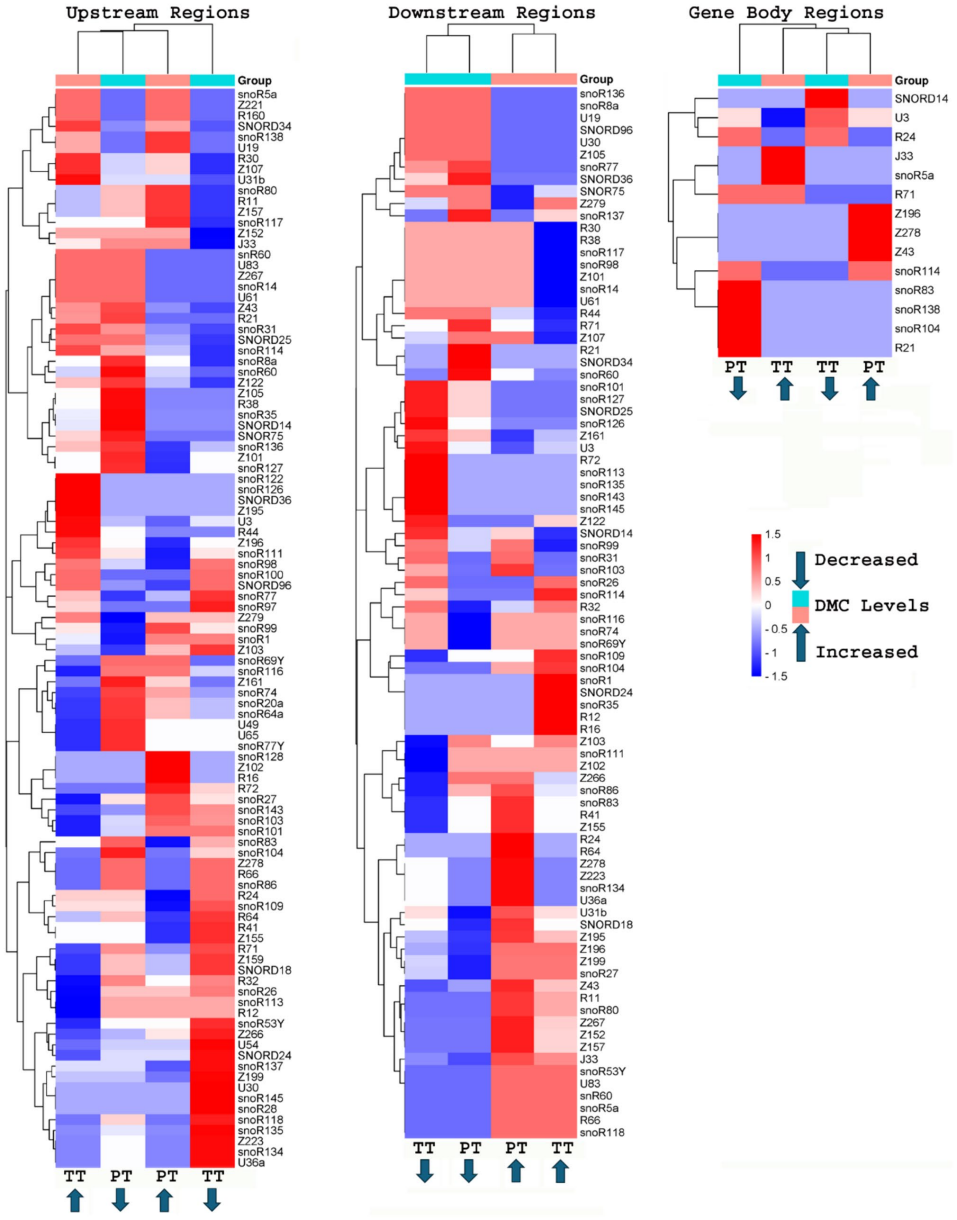
A cluster heatmap of snoRNA genes and ^m^CG + ^m^CHG-DMCs with increased or decreased methylation levels. TT is TK (ungraft) vs homograft [TM-1—TM-1], PT is TK vs heterograft [Pima 3–79—TM-1]. DMC: differentially methylated cytosine

The heatmap of individual snoRNA genes was based on a matrix constructed by dividing the number of DMCs-with increased or decreased methylation levels by the copy number of the snoRNA gene. Comparison studies of ungraft TK vs PT in GB regions revealed that R21, snoR104, snoR138, and snoR83 had higher numbers of ^m^CG + ^m^CHG-DMCs with decreased methylation levels while Z43, Z278 and Z196 had higher numbers of ^m^CG + ^m^CHG-DMCs with increased methylation levels. On the other hand, comparison studies of ungraft TK vs TT in GB regions showed that J33 and snoR5a had higher numbers of ^m^CG + ^m^CHG-DMCs with increased methylation levels while SNORD14 had higher numbers of ^m^CG + ^m^CHG-DMCs with decreased methylation levels (Fig. 3). These results revealed that snoRNAs R21, snoR104, snoR138 and snoR83 in TT, and J33 and snoR5a in PT significantly demethylated by the effects of rootstocks.

As in GB regions, certain snoRNAs in UPS and DWN regions showed significant methylation differences. In UPS regions, U36a, snoR134, Z223, snoR135, snoR118, snoR28, snoR145, U30, Z199, snoR137, SNORD24, U54, Z266, snoR97 and snoR53Y had higher numbers of ^m^CG + ^m^CHG-DMCs with decreased methylation levels, while U31b, snoR122, snoR126, SNORD36, Z195, U3 and R44 had higher numbers of ^m^CG + ^m^CHG-DMCs with increased methylation levels in ungraft TK vs TT. Comparison studies of ungraft TK vs PT in UPS regions showed that snoR60, snoR104, Z105, Z161, R38, snoR35, and SNORD14 had higher numbers of ^m^CG + ^m^CHG-DMCs with decreased methylation levels while snoR128, Z102, R16 and R72 had higher numbers of ^m^CG + ^m^CHG-DMCs with increased methylation levels (Fig. 3).

Comparison studies showed that R16, R12, snoR35, SNORD24, snoR1, and snoR114 had higher numbers of ^m^CG + ^m^CHG-DMCs with increased methylation levels while snoR126, R72, snoR145, snoR143, snoR135 and snoR113 had higher numbers of ^m^CG + ^m^CHG-DMCs with decreased methylation levels in ungraft TK vs TT in DWN regions. Studies revealed that U36a, snoR134, Z223, Z278, and R64 had higher number of ^m^CG + ^m^CHG-DMCs with increased methylation levels, while snoR60, SNORD34, R21, snoR137, SNORD36, R24 had higher numbers of ^m^CG + ^m^CHG-DMCs with decreased methylation levels in ungraft TK vs PT in DWN regions (Fig. 3). Certain snoRNA genes showed differential methylation levels either increase or decrease depending on the gene element they were encoded. For instance, in UPS regions, U36a, snoR134, and Z223 had a higher number of ^m^CG + ^m^CHG-DMCs with decreased methylation levels, while these same snoRNAs in DWN regions showed increased methylation levels.

### Grafting effects on DNA sequence variations

**Fig. 4:**
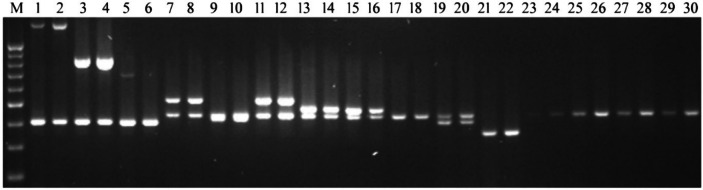
Representative images of DNA analysis showed unaltered DNA sequences between grafts and controls. M: PCR size markers ranged from 200 to 1000 bp. Lanes with odd numbers showed PCR amplified products of TT [TM-1—TM-1] and lanes with even numbers represented PCR amplified products of PT: [Pima 3–79—TM-1] using primer pairs KM16-KM30 with internal primers of INT01 or INT02 listed in Table 1. Primer pair INT1 was internally used with KM16-KM18, and primer pair INT02 was internally used with KM19-KM30

To investigate whether grafting caused any DNA alterations or transfer in the genome of seed samples, DNA samples were obtained from TT, PT and TK, and were amplified in PCR studies using the KM primers coamplified with internal primers of INT01 and INT02 listed in Table 1. Primer pairs, INT01 and INT02 were designed to use as internal controls from reference genome sequences of TM-1 and Pima 3–79. Since KM primer pairs flanked at least one *Msp*I recognition site, *Msp*I was used to digest PCR products to identify any DNA alterations. Screening studies revealed that when primer pairs KM16 to KM18 were internally used with INT01 and primer pairs KM19 to KM30 were internally used with INT02, successful amplifications were obtained (Fig. 4). However, there were no successful amplifications when primer pairs KM24, KM27-KM30 and INT02 were used with internal primer pairs. On the other hand, primer pair KM26 produced amplicon but failed to amplify the product of primer pair INT02. Based on the amplification and digestion studies, the results clearly revealed that there were no alterations in the number of bands between TT, PT, and TK. However, previous reports regarding DNA changes in genomic and plastid DNA were reported in the literature. For instance, plastid DNA was exchanged between the chimeric tissues of two tobacco plants by grafting (Stegemann and Bock 2009) and the entire nuclear genome was transferred between cells of two *Nicotiana* species.

## Discussion

Results revealed that improvement in SVI and 100SW were associated with grafting in cotton. A considerable number of previous studies reported that grafting caused alterations in some phenotypic traits. These examples include increased fruit size and rind thickness in watermelon (Rouphael et al. 2008; Garcia-Lozano et al. 2020), enhanced vigor in eggplant (Cerruti et al. 2021). Grafting induced physiological effects included cold-tolerance, tomato spotted wilt virus, cucumber mosaic virus, and late blight resistance gains in tomatoes (Catoni et al. 2013; Spano et al. 2017; Li and Zhao 2021), seed oil content, gossypol content, pigment density in cotton (Karaca and Ince 2023b; Ye et al. 2023). However, phenotypic alterations have not always been associated with phenotypical changes in grafts. For instance, no phenotypic differences were detected in tomato (Wang et al. 2019).

It is important to note that altered SVI and 100SW traits were obtained from progenies of grafts and controls. Alteration of phenotypes and physiology in graft plants themselves is expected since the scion and rootstock fuse in a compatible match to form combined physiology in a single plant. For instance, a considerable number of physiological processes were influenced in scion, such as vigor or biomass accumulation, fruit quality (Melnyk et al. 2015; Hu et al. 2016). Since altered SVI values were obtained from the seeds of progenies, directed variations were considered heritable. Literature existed indicating grafting directed heritably inherited phenotypes. For instance, Cao et al. (2016) reported stably inherited leaf shape variations in grafts of tuber mustard and red cabbage. Liu et al. (2023a, b) reported heritable DNA methylation changes in the leaf shape and gene expressions, which were maintained for five generations in *Brassica*. These results and many other findings suggested that grafting could cause variations in phenotypic traits (Wu et al. 2013; Cao et al. 2016; Liu et al. 2023a, b). Grafting induced modifications were originally thought to only affect an individual during their lifespan and were not perceived to impact the offspring. In 1956, these beliefs were challenged, and ideas that traits induced by the environment could be inherited began to be discussed (Waddington 1956).

It was noted that the direction of methylation changes, either increase or decrease, in grafts showed variations in the literature depending on methods of grafting, method of identification, coverage and sequencing depth and species of plants (Condon et al. 2018; Jeynes-Cupper and Catoni 2023; Liu et al. 2023a, b). For instance, Cerruti et al. (2021) reported a genome-wide decreased methylation context of heterograft eggplant (*Solanum melongena* L.) grafted onto *Solanum torvum* Swartz using WGBS, while Avramidou et al. (2015) observed a significant increase of global DNA methylation in cucumber and melon scions using the methylation-sensitive amplified polymorphism. Literature is being accumulated with the increased number of studies reporting inherited methylation changes in progenies of grafts. Wu et al. (2013) reported that grafting in three *Solanaceae* species caused extensive alterations of DNA methylation inherited from sexual progenies, with some sites showing further alterations or revisions. Perrin et al. (2020) found significant DNA methylation changes caused by grafting in apple (*Malus domestica*). The authors found that the majority of DNA methylation patterns from the mature donor tree were transmitted to newly grafted plants. In tomato and *Arabidopsis*, the vigorous phenotype induced by a mutation in *MSH1* passed through grafting via siRNAs and inherited in the offspring of wild-type scions (Kundariya et al. 2020). Transgenerational transmissions of methylation changes into the progeny were also reported in other studies (Erdmann and Picard 2020; Boquete et al. 2021). However, there were limited studies on the methylation status of snoRNA genes in grafting. Based on the WGBS data of this study, it was observed that there was a considerable amount of methylation alterations among TT, PT and ungraft TK in DMCs of snoRNA genes. Results revealed that the majority of snoRNA genes showed higher numbers of ^m^CG + ^m^CHG-DMCs with increased methylation levels in PT, while there were higher numbers of ^m^CG + ^m^CHG-DMCs with decreased methylation levels in TT.

The roles of snoRNA genes vary from modification, processing, and assembly of ribosomal RNAs to processing of small nuclear RNAs (snRNAs) and other cellular RNAs (Li et al. 2005a, b; Chen et al. 2021; Wu et al. 2021). The snoRNA genes studied represented both classes: C/D box and H/ACA box. C/D box snoRNAs guide 2′-O-ribose methylation while H/ACA box snoRNAs direct isomerization of uridine to pseudouridine (Ψ) in the post-transcriptional processing of rRNA (Li et al. 2005a, b; Chen et al. 2021; Wu et al. 2021). Among the snoRNA genes studied, the snoR71 gene was the most abundant snoRNA family, which belongs to the C/D box class and functions in the modification of other snRNAs (Galardi et al. 2002). GB regions of snoR71 had higher numbers of ^m^CG + ^m^CHG-DMCs with decreased methylation levels in PT while it had higher numbers of ^m^CG + ^m^CHG-DMCs with increased methylation level in TT (Fig. 3). UPS and DWN regions of the snoR71 gene in PT were less methylated than in TT. Reduced methylation of the snoRNA gene in UPS would enhance its expression levels in PT. Enhanced expression levels would associate with increased ribosome processing, methylation, and assembly. Ribosome biogenesis is a key process in all eukaryotic cells that requires hundreds of ribosome biogenesis factors to build mature ribosomes (Fernandes et al. 2023).

snoRNA genes; U36 (C/D class), snoR134 (H/ACA class), and Z223 (C/D class) showed considerable differences in the numbers of ^m^CG + ^m^CHG-DMCs not only between grafts but among snoRNA gene elements. These snoRNA genes had increased methylation levels in UPS regions but had decreased methylation levels in DWN regions in PT while they showed no methylation differences in TT. This indicates that the effects of grafting had different responses among snoRNA genes and within the gene elements. On the other hand, some snoRNA genes showed similar, either increased or decreased responses to grafting. For instance, snoR60, snoR118, U83 and snoR53Y had similar responses of methylation levels in TT and PT compared to ungraft control in DWN regions. Some other RNA genes such as snoR5a, J33, U31b showed increased or decreased methylation levels in TT, PT, and gene elements. Figure 3 showed more examples of snoRNA methylation differences indicating that some snoRNA genes with statistically significant different methylation levels could be potentially used as biomarkers to identify specific events such as grafting. For instance, Wang et al. (2024) reported that expressions of some noncoding RNAs were related with fiber strength, fiber uniformity and fiber fineness in cotton.

Methylation level differences among snoRNA genes and within the UPS, GB and DWN regions would impact on not only the expression of snoRNA themselves but also the expression of other genes which would be indirectly affected the expression of snoRNA genes. Changes in the trans-generationally inherited methylation levels of snoRNA genes directed by grafting are important since plant genomes contain a considerable number of snoRNA genes. It is well known that snoRNAs regulate essential genes for plant growth and development, and plant adaptation to different habitats or extreme environments. Chen et al. (2022) showed that DNA methylation alterations affected the interaction with histone acetylation which played significant roles in fiber cell initiation of *G. hirsutum*. Chen et al. (2021) showed that expressions of 13 snoRNAs were positively correlated with latex production. Xiao et al. (2022) reported that certain snoRNA genes were associated with chromatin in a tissue‐specific manner during somatic cell reprogramming in rice and played a role in improving rice yield. Studies of Chen et al. (2018) revealed that plant‐specific regulator histone deacetylase (HDAC) controls plant snoRNA gene transcription by associating with the promoter regions of the U14 gene and repressing its expression. Zheng et al. (2019) found that some snoRNAs played regulatory roles by regulating the stability and activity of rRNAs and splicing small nuclear RNAs under drought conditions.

## Conclusions

Cotton contained more snoRNA genes compared to rice, maize, and some other genomes. The higher occurrence of snoRNA genes and their significance roles in cotton make them good targets to study the locus specific methylation changes in homografting and heterografting experiments. DNA cytosine methylation levels of snoRNA genes in the context of ^m^CG were always higher than ^m^CHG and ^m^CHH in seed genomic DNAs obtained from offspring of grafts and control. Statistically significant DMC differences among gene elements (UPS, GB, DWN), TT, PT, and snoRNA genes were identified in the absence of DNA sequence alterations. The findings of the present study also provided good examples of the importance and effects of grafting directed DNA methylation of snoRNA genes in two trans-generationally inherited traits seedling vigor index and 100 seed weight that are potentially useful in breeding schemes to consistently increase crop production in cotton. However, further studies are required to investigate the molecular mechanisms associated with the change in trans-generationally inherited methylation levels of snoRNA and other genes directed by grafting. Based on the results of this study, we speculate that selection based on differential methylation of snoRNA genes could be useful for breeding studies to obtain higher adaptability, yields, and quality.
